# The Application of *in silico* Methods for Prediction of Blood-Brain Barrier Permeability of Small Molecule PET Tracers

**DOI:** 10.3389/fnume.2022.853475

**Published:** 2022-03-25

**Authors:** E. Johanna L. Stéen, Danielle J. Vugts, Albert D. Windhorst

**Affiliations:** Amsterdam Neuroscience, Department of Radiology and Nuclear Medicine, Amsterdam UMC, Vrije Universiteit, Amsterdam, Netherlands

**Keywords:** central nervous system, PET tracer, blood-brain barrier, *in silico* prediction, QSAR models

## Abstract

Designing positron emission tomography (PET) tracers for targets in the central nervous system (CNS) is challenging. Besides showing high affinity and high selectivity for their intended target, these tracers have to be able to cross the blood-brain barrier (BBB). Since only a small fraction of small molecules is estimated to be able to cross the BBB, tools that can predict permeability at an early stage during the development are of great importance. One such tool is *in silico* models for predicting BBB-permeability. Thus far, such models have been built based on CNS drugs, with one exception. Herein, we sought to discuss and analyze if *in silico* predictions that have been built based on CNS drugs can be applied for CNS PET tracers as well, or if dedicated models are needed for the latter. Depending on what is taken into account in the prediction, i.e., passive diffusion or also active influx/efflux, there may be a need for a model build on CNS PET tracers. Following a brief introduction, an overview of a few selected *in silico* BBB-permeability predictions is provided along with a short historical background to the topic. In addition, a combination of previously reported CNS PET tracer datasets were assessed in a couple of selected models and guidelines for predicting BBB-permeability. The selected models were either predicting only passive diffusion or also the influence of ADME (absorption, distribution, metabolism and excretion) parameters. To conclude, we discuss the potential need of a prediction model dedicated for CNS PET tracers and present the key issues in respect to setting up a such a model.

## Introduction

**Figure 1:**
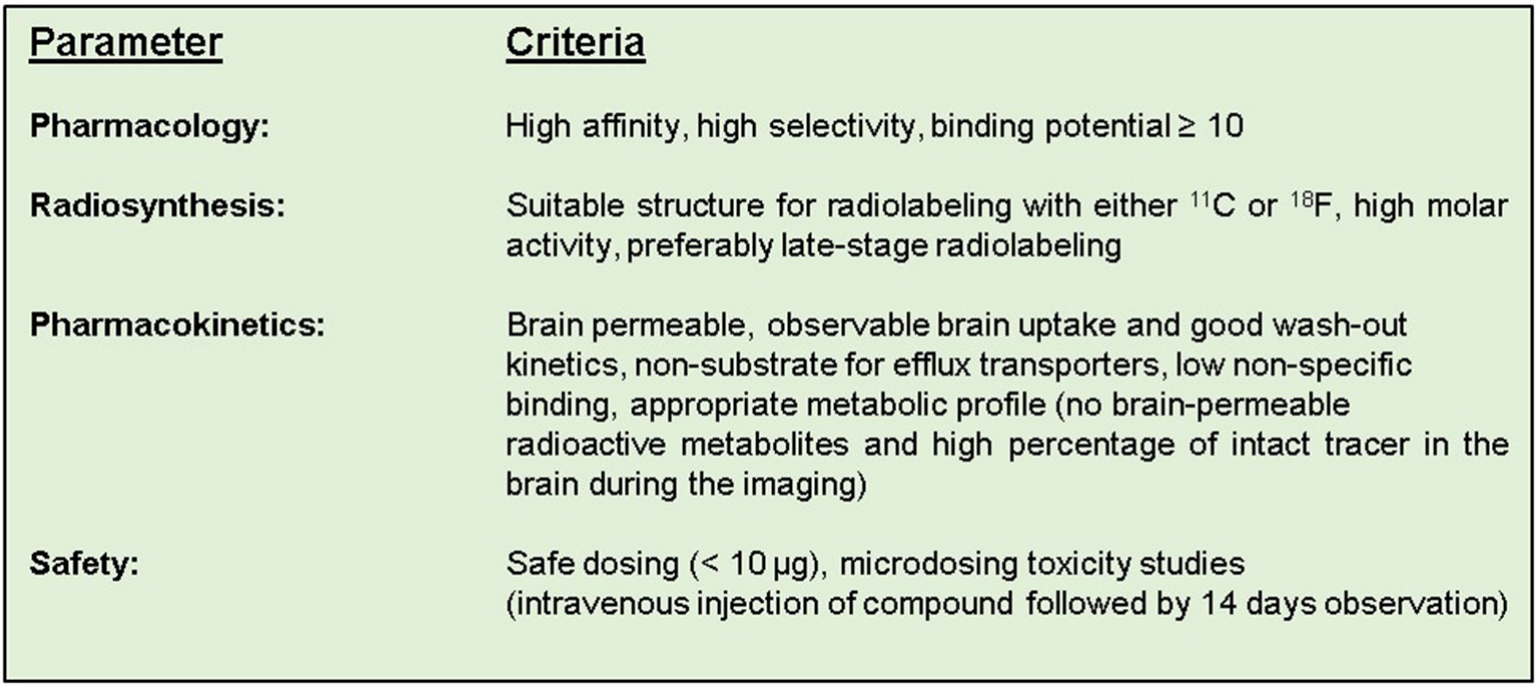
The suggested ideal criteria for a successful CNS PET tracer.

Neurodegenerative and neurological diseases, such as Alzheimer's disease, schizophrenia, Parkinson's disease and multiple sclerosis affect millions of people worldwide and together they comprise one of the world's most important health challenges (1). Therefore, it is of utmost importance to get a better understanding of the mechanisms of these diseases at a molecular level. In this way current diagnosis, prognosis and treatments can be improved or even replaced by better alternatives. Positron emission tomography (PET) allows us to study these diseases and accelerate drug development, as well as to explore both new treatment opportunities and targets (2, 3). However, as for traditional central nervous system (CNS) drug discovery, the development of PET tracers for brain targets is a challenging task and there is a high attrition rate. A successful CNS PET tracer has to fulfill several criteria (Figure 1) in regard to its pharmacology, structure, pharmacokinetics and safety/toxicity (4–7). First of all, the tracer has to show a high affinity and selectivity toward its intended target. Preferably, the affinity (indicated with a dissociation constant *K*_d_ or an inhibitory constant *K*_i_) should be in the low single digit nM to sub-nM range. Secondly, the binding potential (*BP*), defined as *B*_max_/*K*_d_, ought to be equal or higher than 10. Here, *B*_max_ represents the maximum concentration of target binding sites. Sometimes the term *B*_avail_ is used instead of *B*_max_ for *in vivo* purposes. The former is defined as the maximum concentration of available target binding sites and used for *in vivo* purposes, since for conditions *in vivo* not all sites will be available for tracer binding due to occupancy by endogenous ligands (8, 9). The value of *BP* gives a rough estimation of how likely a target can be imaged by a specific tracer. Thus, the lower expression level the intended target has, the higher affinity (a *K*_d_ value in the sub-nM range) is needed for adequate imaging. A *BP* ≥ 10 should not be considered as a strict threshold, but as a rule of thumb. For example, an important exception is [^11^C]raclopride, which has a *BP* of 5.4 and is still an excellent tracer for imaging of the dopamine receptor D2 (9). Even though the *BP* gives an estimation of the potential image quality for a specific tracer/target pair, the image quality is not solely dependent on the signal arising from target-bound tracer (specific binding). The background signal arising from non-specific binding also plays a major role. Thus, low non-specific binding (NSB) is preferred to allow sensitive imaging. Previously, NSB was thought to mainly correlate to descriptors related to lipophilicty, e.g., the logarithmic partition coefficient (log*P*) and the logarithmic distribution coefficient at physiological pH (log*D*_7.4_) of the tracer. However, this correlation is not perfect and other descriptors, such as the charge state of a compound and the degree of ionization at physiological pH have been reported to influence as well (10–12).

From a chemistry point of view, the structure of the PET tracer has to allow for late-stage radiolabeling with either carbon-11 or fluorine-18 in high molar activity. Moreover, a suitable metabolic profile of the tracer is required with no or minimal amounts of radiolabeled metabolites taken up by the brain. Ideally, the tracer should degrade outside the brain to less lipophilic metabolites that cannot enter the brain. Possible metabolites that could be formed should be taken into account already during the selection of radiolabeling approach. Besides being metabolically stable to reach its intended target *in vivo*, the tracer must also be able to cross the blood-brain barrier (BBB). The BBB is playing a critical role in protecting the brain against compounds circulating in the blood stream by acting as a significant obstacle into the CNS (13). To make passage even more problematic, the BBB also contains numerous of efflux transporters, such as P-glycoprotein (P-gp), the breast cancer resistant protein (BCRP) and the multidrug-resistance proteins 1–3 and 5 (MDR1–3, 5). The main task of these proteins is to transport exogenous compounds out from the brain (14, 15). Obtaining tracers with a sufficient BBB-penetration is often considered the major hurdle during the development phase. An estimation is that only 2% of small molecules are able to cross the BBB (16). Therefore, during tracer development special attention should be given to screen for and design structures that are BBB-permeable. Computational methods for predicting brain uptake based on descriptors derived from the molecular structure can guide and accelerate this step in the development phase. The research regarding *in silico* predictions for brain uptake is extensive and numerous models have been reported over the years. Naturally, all reported models except one have been based on CNS drugs and not CNS PET tracers. Most often PET tracers are simply radiolabeled analogs of already reported compounds showing affinity for the specific target to be studied. However, due to significant differences in administered dose (microgram *vs*. milligram), as well as the route of administration (intravenous *vs*. oral) between CNS PET tracers and CNS drugs, the covered space in ADME(T) (absorption, distribution, metabolism, excretion, and toxicity) properties often differ. As a result, *in silico* predictions that take such parameters into account and are based on data derived from CNS drugs, may not predict CNS PET tracers as accurately. In case of only predicting passive diffusion, which is merely a compound's fusion into the lipid membrane and a non-saturable mechanism, the favored property range for tracers and drugs should not be different (17, 18). This, in turn, is further supported by the fact that radiolabeling of CNS drugs is frequently used to study brain penetration and biodistribution during the development phase. This should not be confused with the assessment if a compound entering the brain *via* passive diffusion will in fact have a high enough brain uptake to be a successful imaging agent (18).

We will discuss if a dedicated *in silico* model for CNS PET tracers is needed or if a model built on a CNS drug dataset can be used for predicting brain uptake of CNS PET tracers as well. To address this, we will provide a short historical overview of *in silico* prediction models for BBB-permeability, and what is needed in terms of establishing and validating such models. Furthermore, we have highlighted a few selected contributions with the aim of showing different types of computational methods used. In addition, we have included a comparison of eight different prediction models and classification rules using a combination of previously reported CNS PET tracer datasets. Finally, we will end with discussing the potential need of an *in silico* BBB-permeability prediction dedicated to CNS PET tracer development, as well as presenting the possible issues with developing such a model.

## Datasets for *in silico* Modeling to Assess Brain Uptake

In order to build a prediction model, a dataset of sufficient size and quality is needed. The dataset has to include both positives and negatives for what the model is going to predict. In theory, experimental values used to define positives and negatives can be retrieved based on either *in vitro* or *in vivo* measurements. In the case of *in silico* models for BBB-permeability, positives will be compounds that can cross the BBB, whereas negatives would be compounds that cannot. Evidently, *in vivo* measurements are always preferred and for BBB-permeability there are two types of data that can be used for defining positives and negatives; (A) numerical, and (B) categorical. The most commonly used numerical data is the logarithmic brain:plasma concentration at steady-state (log*BB*) as displayed in Equation (1). Several log*BB* values have been collected and combined to create large datasets (19).


(1) $$\text{logBB}\;=\text{ log}\frac{{\text{C}}_{\text{brain}}}{{\text{C}}_{\text{blood}}}$$

Another descriptor for BBB-permeability is the logarithmic permeability surface-area product, expressed as log*PS*. *PS* (measured in the unit mL/min/g brain) is obtained *via in situ* brain perfusion studies and by using the Renkin-Crone equation (Equation 2), where *F* is the cerebral blood or perfusion flow rate and *K*_*in*_ is the unidirectional transfer constant. The latter can be derived from Equation (3), in which *Q*_*br*_ is the concentration, corrected for the vascular volume, of compound in the brain, *C*_*pf*_ is the concentration of compound in the perfusion fluid and *T* is the perfusion time (20–24). In contrast to log*BB*, which is a measurement at steady-state, log*PS* is a measurement of the initial permeability rate and can be seen as a brain pharmacokinetic value. Log*PS* is more informative than log*BB*, but less used since it is labor-intensive and has a low-throughput (20, 21). Because of this, not much data is available, which limits the use of these types of datasets for establishing an accurately trained and validated *in silico* prediction model. In addition, when using numeric data an appropriate threshold for defining positives and negatives is required.


(2) $$\text{PS}\;=\;-\text{Fln}\left(1\;-\;\frac{{\text{K}}_{\text{in}}}{\text{F}}\right)$$

(3) $${\text{K}}_{\text{in}}\;=\;\left(\frac{{\text{Q}}_{\text{br}}}{{\text{C}}_{\text{pf}}}\right)/\text{T}$$

With regard to categorical data a compound is classified as CNS+ or CNS–, based on its CNS activity, hence this type of data has two assumptions. Firstly, for CNS+ compounds, they certainly cross the BBB (or potentially an active metabolite of the parent compound, e.g., in the case of prodrugs), but the mechanism of action can vary among the compounds including passive diffusion, carrier-mediated transport or receptor-mediated transcytosis. The CNS+ classification can also be misleading in cases when the compound has a low BBB permeability, but still a high potency. Secondly, the classification implies that the CNS– category does not cross the BBB, which is not necessarily true. Some of these compounds may still cross the BBB, but they do not show CNS activity. The lack of CNS activity can be a result of no interaction with a CNS target, rapid metabolism or the compounds are substrates for efflux transporters (19). To sum up, the use of categorical data makes it easier to find a CNS+ compound than identifying a CNS– compound. Often, a combination of numerical and categorical datasets is used, in which the numerical data have been further defined with a threshold for classifying CNS+ and CNS–, respectively. Furthermore, in categorical datasets, P-gp substrates are sometimes included. Thus, predictions based on such dataset will not only account for passive diffusion through the BBB, but also the potential contribution of active transport arising from P-gp.

The dataset that is applied for creating the model is called a training set. However, a properly established model should also have its performance validated. Shortly, the validation gives an indication on how well the model can classify a dataset. The actual validation is performed by screening an additional dataset, called the test set. The latter should contain different compounds than the training set. The performance of the model is assessed by several commonly used statistical parameters as outlined in Table 1 (25–27). Mind that even though accuracy (*ACC*) is one of the most commonly used statistical parameters to evaluate classification systems, it is not a proper measurement when working with imbalanced dataset. Instead Matthew's correlation coefficient (MCC) and the corrected classification rate (CCR) are more robust parameters to use for these types of datasets (27, 28).

**Table 1:** Commonly used statistical parameters for evaluating the performance of a classification model.

**Parameter**	**Definition**	**Formula**
Accuracy (*ACC*)	The total fraction of compounds correctly classified in the dataset	$ACC\;=\;\frac{TP\;+TN}{n}$
Error rate (*ER*)	The total fraction of compounds incorrectly classified in the dataset	$ER\;=\;\frac{FP\;+FN}{n}\;=\;1\;-ACC$
Sensitivity (*Se*)	The ability of the model to find true positives (*TP*)	$Se\;=\;\frac{TP}{TP\;+\;FN}$
Specificity (*Sp*)	The ability of the model to find true negatives (*TN*)	$Sp\;=\;\frac{TN}{TN\;+\;FP}$
False negative rate (*FNR*)	Fraction of positives incorrectly classified as negatives	$FNR\;=\;\frac{FN}{TP\;+FN}\;=\;1\;-Se$
False positive rate (*FPR*)	Fraction of negatives incorrectly classified as positives	$FPR\;=\;\frac{FP}{FP\;+TN}\;=\;1\;-Sp$
Positive predictive value (*PPV*)	The fraction of *TP* out of the total amount of instances classified as positives. The *PPV* is also known as precision	$PPV\;=\;\frac{TP}{TP\;+FP}$
Negative predictive value (*NPV*)	The fraction of *TN* out of the total amount of instances classified as negatives	$NPV\;=\;\frac{TN}{TN\;+FN}$
Matthew's correlation coefficient (*MCC*)	*MCC* is also known as the mean square contingency coefficient (ϕ). It is a measure of association between two binary variables	$MCC\;=\;\frac{TP\;\times TN\;-FP\;\times FN}{\sqrt{\left(TP\;+FN\right)\left(TP\;+FP\right)\left(TN\;+FN\right)\left(TN\;+FP\right)}}$
Corrected classification rate (CCR)	*CCR* is a measure of the correct classifications in the dataset	$CCR\;=\;\frac{1}{2}\left(\frac{TN}{N}\;+\;\frac{TP}{P}\right)$

*TP = number of true positives (CNS+ compounds in the dataset classified correctly); TN = number of true negatives (CNS– compounds in the dataset classified correctly); n = total number of compounds in the dataset (N + P); FP = number of false positives (true CNS– compounds classified as CNS+ by the model); FN = number of false negatives (true CNS+ compounds classified as CNS– by the model); N = total number of negatives (CNS– compounds) in the dataset; P = total number of positives (CNS+ compounds) in the dataset*.

## Overview of *in silico* Models for Prediction of Blood-Brain Barrier Permeability

Already at the turn of the twentieth century, Meyer and Overton discovered, in two independent studies, a correlation between the partition coefficient in olive oil:gas and potency of common anesthetic agents. The more lipophilic an anesthetic was (i.e., higher partition coefficient), the greater potency it had. This later became recognized as the Meyer-Overton correlation for anesthetics (29–31). From there on research was directed toward understanding the composition and biophysical properties of the BBB along with studying the BBB-permeability of different charged and uncharged compounds. Not until around the 1970–80's the focus was moved toward investigating correlations and relationships between different physicochemical properties of compounds and how changes in these influence permeability and brain exposure. Lipohilicity has long been recognized as the key property influencing permeability, thus a heavy reliance has been placed on optimizing only this descriptor to increase permeability. However, with better understanding of the physiology of the BBB it became apparent that other properties play a part as well. Pioneering work by Levin et al., describes the influence of size. The authors observed a relationship between BBB-permeability, lipophilicity and molecular weight (MW). The permeability was improved with increasing log*P* (experimentally measured in 1-octanol:water) for compounds having a MW <400 g mol^−1^ (32). Another important study, conducted by Young et al., showed the effect of the hydrogen bonding potential (33). In fact, the rate-limiting step when a compound crosses the BBB is suggested to be the hydrogen bonding interactions of the compound to the hydrophilic part of the lipids in the BBB (34). Molecular descriptors encoding for hydrogen bonding information are, e.g., hydrogen bond acceptors (HBAs), hydrogen bond donors (HBDs) and (topological) polar surface area ((T)PSA) (35).

With more experimental data collected and correlations investigated for numerous molecular descriptors (both experimental and computed ones), groups started to set up prediction models. The simplest type of prediction models are “rules of thumb”. These give a rough and quick guidance for what property range has the highest probability to favor brain permeability. After Lipinski's rule of five (36) for orally administered drugs, a set of rules specifically for CNS drugs were derived stating that a CNS drug should preferably have the following properties: (A) MW ≤ 400 g mol^−1^; (B) computed logarithmic partition coefficient (Clog*P*) ≤ 5; (C) HBAs ≤ 7; (D) HBDs ≤ 3 (35). In contrast, van de Waterbeemd et al. suggested rules in which the upper limit for MW is 450 g mol^−1^ to favor brain permeation, while PSA should be < 90 Å^2^ and log*D*_7.4_ in the range of 1–4 (37). Following this, Kelder et al. narrowed the PSA range a bit further to 60–70 Å^2^, as a result of studying the PSA contribution for a larger dataset involving 776 CNS drugs (38). Norinder and Haeberlein derived additional rules based on the ones mentioned above and applied an additional dataset reported by Clark (39, 40). Their first rule states that if the sum of nitrogen and oxygen atoms (*N*+*O*) in a molecule is five or less, the compound has a higher chance of brain permeation. The authors also showed that this simple rule with other datasets could predict as accurate as more complex models. Next, they stated a second rule that predicts if log*P*-(*N*+*O*) of a specific compound is positive, then its log*BB* is also positive and the compound has a high probability to cross the BBB. Both of these quick rules of thumb were able to predict the applied datasets with high *ACC* (0.85 and 0.92, respectively) (39). It should be noted that this is the *ACC* from the datasets used to find the relationships. The rules were only validated with one additional dataset comprising 29 compounds. Although, the *ACCs* were again relatively high (0.72 and 0.79, respectively), a validation with a larger dataset would have been preferred. However, at that time there was limited access to such a dataset. To follow this, Hitchcock and Pennington reported a set of rules based on reviewing properties of, at the time, available CNS drugs. The suggested property thresholds were: Clog*P* = 2–5, computed logarithmic distribution coefficient at physiological pH (Clog*D*_7.4_) = 2–5, MW < 500 g mol^−1^, PSA < 90 Å^2^, HBD < 3 (41). A few years later, Manallack reviewed the literature to study the distribution of the acid-base dissociation constant (p*K*a) among 528 drugs. From these he made a subset consisting of 174 CNS and 408 non-CNS drugs for which it was revealed that CNS drugs rarely have acidic p*K*a values below 6, whereas no CNS drugs had basic p*K*a values above 10.5 (42).

A more extensive, and more recent, property profile for CNS+ *vs*. CNS– was created by Ghose et al., who analyzed simple physicochemical properties (and no ADME parameters) of a categorical dataset comprising 317 CNS and 626 non-CNS drugs. This high number of non-CNS drugs, actually more than the number of CNS drugs, is an advantage of this work. They derived a few guidelines that can be applied in lead optimization toward getting a BBB-permeable structure. In summary, the analysis resulted in the following guidelines: (A) TPSA < 76 Å^2^ (25–60 Å^2^); (B) at least one (1 or 2, including 1 aliphatic amine) nitrogen atom; (C) < 7 (2–4) linear chains outside of rings; (D) < 3 (0 or 1) polar hydrogen atoms; (E) volume of 740–790 Å^3^; (F) solvent accessible surface area of 460–580 Å^2^; (G) positive QikProp parameter CNS (43). The latter is a software-specific function for ADME prediction in the molecular modeling software package Schrödinger®, which limits its application for general use.

More advanced than applying simple rules of thumb is to predict an actual value of log*BB*. The first purely computational approach for this purpose was that of Kansy and van de Waterbeemd. Multiple linear regression was carried out on physicochemical property data from 20 compounds to generate Equation (4), with a correlation coefficient (*R*^2^) of 0.84, a root-mean-square error (*RMSE*) of 0.45 and a Fisher value (*F*) of 20 (44). Not surprisingly, the model was not very predictive when applied on another dataset, which is most likely a result of the limited number of compounds used to establish the model. Abraham et al. also developed numerous equations to predict log*BB* using larger datasets and with better performances (39, 45). However, the calculations of the parameters were not straightforward and rather time-consuming. A model relaying on more easily calculated descriptors was set up by Clark. The prediction model (Equation 5) was established with a training set consisting of 57 compounds and it showed good performance (*R*^2^ = 0.89, *RMSE* = 0.35, and *F* = 96). However, it was only validated using two very small test sets comprising 6 and 7 compounds, respectively. On the other hand, the predicted log*BB* was comparable to the experimentally determined ones for most compounds (40). Finally, two log*BB* predictions (Equations 6 and 7) using a more extensive dataset were reported in 2010 by Vilar et al. Equation (6) is based on two descriptors, Clog*P* and TPSA, whereas the second prediction (Equation 7) is based on the same descriptors, but with the addition of the sum of the number of acidic and basic atoms (*a*_*acid*_ and *a*_*base*_, respectively). The training set consisted of 307 compounds all with experimental log*BB* values determined by *in vivo* studies. Both models should be used in the prediction of a new compound since they are set up based on general log*BB* thresholds for CNS+ (log*BB* ≥ 0.3, Equation 6) and CNS– (log*BB* ≥ −1, Equation 7) classification. If the result of the prediction of a tested compound is > 0, the prediction is that its log*BB* is ≥ 0.3 for model 6 and log*BB* ≥ −1 for model 7. The models were later validated using a test set (1,222 CNS+ and 235 CNS– compounds) based on categorical data (46). The authors stated that it does not matter that a categorical dataset was applied in the validation, because CNS activity anyway implies BBB-permeability. Indeed this is true, but as previously mentioned the models may have difficulties finding CNS– compounds. Another drawback is that the method is not really applicable for screening a larger set of compounds. For instance, as a filter in virtual screening campaigns.


(4) $$\text{logBB}=-0.021\left(\pm 0.003\right)\text{PSA}-0.003\left(\pm 0.001\right)\text{Mo}{\text{l}}_{\text{Vol}}+1.643\left(\pm 0.465\right)$$

(5) logBB=−0.0148(±0.001)PSA + 0.152(±0.036)ClogP+0.139(±0.073)

(6) logBB = 0.5159 × ClogP−0.0277 × TPSA−0.3462

(7) $$\text{logBB}\;=\;0.2289\;\times \;\text{ClogP}-0.0326\;\times \;\text{TPSA}-0.5671\times \left({\text{a}}_{\text{acid}}\;+\;{\text{a}}_{\text{base}}\right)\;+\;2.3420$$

**Figure 2:**
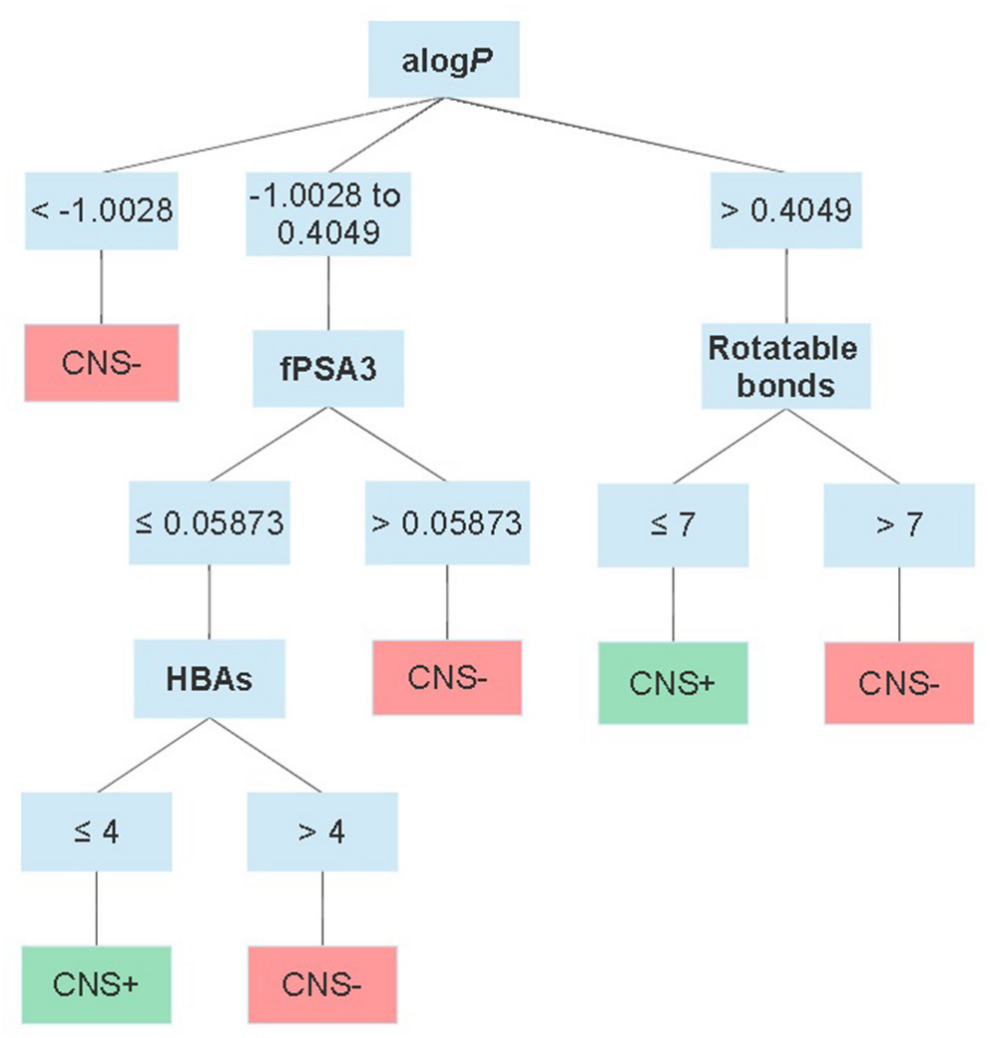
Schematic overview of the CHAID decision tree reported by Suenderhauf et al. displaying the thresholds for each parameter in the classification. alog*P* = the log*P* calculated according to Ghose and Crippen (57); fPSA3 = the charge weighted partial positive surface area divided by the total molecular surface area.

Moving forward to somewhat more advanced prediction models reported the last decade when the available data for setting up a dataset increased and additional descriptors could be applied. Different modeling approaches have been used, e.g., partial least squares regression (47–49), read-across (50) as well as machine learning methods such as decision trees (51), *k*-nearest neighbors (52), neural networks (53), random forests (27), and support vector machines (52, 54–56). Most of them predict log*BB* or just classify compounds as CNS+ or CNS–. However, Suenderhauf et al. reported prediction based on log*PS* with high CCR (~0.90) and MCC values around 0.80. This is one of the few reported models based on only log*PS* data. They built two decision tree models, which were induced for suitable splitting using two paradigms, Chi-squared automatic interaction detector (CHAID) and the classification and regression tree algorithm (CART). The dataset used for establishing the models were based on a dataset of 120 compounds with known log*PS* values. The dataset was further categorized as CNS+ if log*PS* ≥ −2 (*n* = 65) and as CNS– in case of log*PS* ≤ −3 (*n* = 55). To achieve better separability during the training of the model, compounds in the range in between the two cut-off thresholds (−2.1 and −2.9) for CNS+ and CNS– were excluded from the dataset. A 10-fold cross-validation strategy was used to evaluate the models. In this way, the dataset was randomly split into 10 subsets. Out of these, nine subtest were combined and used to train each model and the remaining one was used during validation. This process was repeated 10 times, until all subsets had been used for training and validation. The applied descriptors in the two different models differed, but they both included contributors to lipophilicity, size and charge in the prediction. Figure 2 shows an overview of the decision tree built with CHAID with the splitting criteria outlined. Notably, the threshold for log*P* contribution in these models was lower compared to other prediction models and guidelines. The authors suggest this is an indication of active transport involvement, since compounds suspected of being actively transported were not excluded from the dataset (51). However, it is not stated how many compounds this actually concerns and if it is active influx and/or efflux. In addition, with regard to the limited dataset and the fact that this conclusion is based on the contribution of one property it may be misleading.

Succeeding in preparing a dataset with high quality and a proper size is a constant challenge. As already stated, ~98% of all small molecules are estimated to not cross the BBB. Therefore, if a dataset for validation should capture the reality, only 2% of the compounds in there should be able to cross the BBB. Martins et al. tried to address this issue in 2012 by using Bayesian statistics to create an unbiased dataset for model training and validation. After screening the literature, they created a dataset of totally 2,053 compounds, 1,570 CNS+ and 483 CNS– compounds. The dataset contained both numerical and categorical data. To align the dataset, the numerical data was further categorized as CNS+ in cases of log*BB* ≥ −1 and as CNS– if log*BB* < −1. Compounds with a MW exceeding 600 g mol^−1^ were excluded, resulting in 1970 compounds in the end. In addition, 120 compounds were randomly withdrawn to be used as a test set for validation of the generated models. The authors tried four different descriptor sets, which in turn included numerous different parameters. One set included calculated fingerprints, while the three remaining sets included together 1,701 descriptors [see (27) for details]. Two different machine learning algorithms, i.e., random forests and support vector machines were used to create potential prediction models for further validation. In the end, the best model was a random forests fitted with three of the different descriptor sets. The model showed a high *ACC* (0.95), good *Se* (0.83), and moderate *Sp* (0.71). Although, the *Sp* is comparable to other good-performing BBB-permeability predictions and the *MCC* (0.74) was good (27). The model was made available as a free web-based tool when published (58).

Multiparameter optimization (MPO) desirability tools have found application in BBB-permeability prediction as well. MPO can assess the effects of several descriptors, balanced and weighed with regard to their importance to the overall goal of the tool. In this way, hard cut-offs in property ranges are not needed. In combination with a desirability function, the contribution of multiple components can be transformed *via* function(s) into a single composite score. The transformation functions have defined points indicating desirable and undesirable ranges of the value of the specific component. In 2010, a group at Pfizer set up a MPO tool based on key descriptors for CNS drugs. In total, six descriptors (Clog*P*, Clog*D*_7.4_, MW, TPSA, HBD, and p*K*a of the most basic atom) of 119 CNS drugs and 108 CNS drug candidates were analyzed and aligned with ADMET parameters such as permeability, low P-gp efflux liability, metabolic stability and toxicity. These parameters were assessed in *in vitro* assays. All descriptors were weighed equally and could have a transformed desirability score ranging from 0 to 1. The transformed score of each of these descriptors were summed up to yield the final CNS MPO score, which can be between 0 and 6 (59). Table 2 shows the applied transformation functions, the desirable and undesirable ranges, respectively, for each descriptor.

**Table 2:** The descriptors with desirable/undesirable range indicated and functions used for scoring in CNS MPO and CNS PET MPO (59, 61).

**Descriptor**	**Type of transformation function**	**CNS MPO**		**CNS PET MPO**	
		**Desirable range**	**Undesirable range**	**Desirable range**	**Undesirable range**
MW (g mol^−1^)	Monotonic decreasing	MW ≤ 360	MW > 500	MW ≤ 305.3	MW > 350.5
Clog*P*	Monotonic decreasing	Clog*P* ≤ 3	Clog*P* > 5	Clog*P* ≤ 2.8	Clog*P* > 4.0
Clog*D*_7.4_	Monotonic decreasing	Clog*D*_7.4_ ≤ 2	Clog*D*_7.4_ > 4	Clog*D*_7.4_ ≤ 1.7	Clog*D*_7.4_ > 2.8
TPSA (Å^2^)	Hump function	40 < TPSA ≤ 90	TPSA ≤ 20; TPSA > 120 44.8	44.8 < TPSA ≤ 63.3 TPSA	TPSA ≤ 32.3; TPSA > 86.2
HBD	Monotonic decreasing	HBD ≤ 0.5	HBD > 3.5	HBD ≤ 1	HBD > 2
p*K*a	Monotonic decreasing	p*K*a ≤ 8	p*K*a >10	p*K*a ≤ 7.2	p*K*a > 9.5

*All descriptors were equally weighed*.

When comparing the CNS MPO score with the results from the *in vitro* assays for ADME profiles, a trend was observed that compounds with a higher score had better profiles. Around 77% of the compounds in the CNS drug dataset with a CNS MPO score > 5 displayed a full alignment of all ADME parameters, i.e., high passive permeability, low P-gp liability, appropriate metabolic stability and high cellular viability in the toxicity assay. At the same score threshold, 54% CNS candidates showed full alignment. In the end, the authors suggest that for high probability of successful CNS drug, the CNS MPO score should be > 4 (59). A drawback of this method is that the applied dataset did not include reported unsuccessful CNS drugs (CNS–), only CNS+ and CNS drug candidates. Since some of the candidates had been withdrawn during the drug development process, the authors initially anticipated that they could act as CNS– surrogates and have another distribution in the CNS MPO score. However, this was not the case. Another disadvantage is that the model was not validated with an external dataset. Moreover, there are no lower limits in the desirable space for properties such as Clog*P*, Clog*D*_7.4_, MW and p*K*a. This means that very small and charged compounds will be scored to be in a desirable space as well. On a note, Gunaydin et al. published a probabilistic MPO (pMPO) scoring function that addresses this issue. That model was built on a larger dataset with both CNS+ and CNS– compounds (299 CNS+/366CNS–). The authors also investigated the utility of the scoring method to predict P-gp liabilities. A set of 500 molecules with measured efflux ratios were screened from which it was indicated that pMPO was a fairly good descriptor for P-gp liability as well (60).

A few years later, in 2013, a CNS MPO version for PET tracers was reported. This model was built based on 62 PET tracers validated in the clinic and 15 unsuccessful PET tracers that failed during the late-stage development phase, primarily due to high NSB. When screening the PET dataset in the CNS MPO model, which defines a compound with a CNS MPO score > 4 as a high probability to be successful, 85% of the successful PET tracers were scored above 4 and 15% had a score ≤ 4. For the unsuccessful PET tracers as high as 60% was scored > 4. In contrast, when using the modified MPO with descriptor ranges (Table 2) better for tracers (score for good tracer > 3) the model scored 79% of the successful tracers with a score > 3 and 67% of the unsuccessful tracers with a score ≤ 3. In comparison to the CNS MPO, one additional parameter in the ADME profiling was added, namely NSB. To sum up, increasing the probability of aligning all ADME parameters and increasing the probability of designing a successful PET tracer, the author suggest that the CNS PET MPO score should be > 3 (61). A drawback with this model is that it has not been validated with a test set. However, there is not enough reported CNS PET tracers available, both successful and unsuccessful, for setting up a dataset with adequate size to cover both a training set, as well as a test set. As mentioned for the CNS MPO version, the PET version has the same disadvantage that there are no lower limits in the desirable property space.

In order to address some of the challenges with the reported MPO models, Gupta et al. reported a prediction tool called the BBB score, in which the composite score for each descriptor was transformed *via* stepwise and polynomial piecewise functions. The model comprises five different descriptors; number of aromatic rings, number of heavy atoms, MWHBN (a descriptor based on the MW, HBDs and HBA that can be calculated from HBN/$\sqrt{MW}$, where HBN is the sum of HBDs and HBAs), TPSA and p*K*a at pH 7.4. In contrast to the CNS MPO, the BBB score was trained on a dataset containing both CNS+ (*n* = 270) and CNS– (*n* = 720) compounds. Moreover, the dataset was curated and only passive diffusion was taken into account, meaning compounds with reported active influx/efflux activities were excluded. Validation with an external test set revealed that the models had a high *Se* (0.8) and adequate *Sp* (0.72). The authors screened the same test set in the CNS MPO model as well and a significantly lower *Sp* (0.38 vs. 0.72) was observed (62). Notably, the authors did not state if they used the same software as used by Wager et al. to calculate the different descriptors applied in CNS MPO, which can have a significant impact on the final score. This is especially true for Clog*P* and Clog*D*_7.4_.

To end this section, recent work reported by Jackson et al. should be mentioned. In two conference abstracts the authors have summarized their on-going work toward setting up an *in silico* model built based on successful and unsuccessful CNS PET tracers (75/65). The unsuccessful tracers were further divided into subcategories of tracers that failed due to high NSB (*n* = 25), non-permeable tracers (*n* = 30) and other (*n* = 10). No further details regarding the categorization of the dataset are provided, i.e., if it was solely based on successful/unsuccessful data in humans, what the thresholds for permeable and non-permeable were or if the latter sub-category includes efflux transporter substrates as well (63, 64). Eight different descriptors including Clog*P*, Clog*D*_7.4_, MW, TPSA, p*K*a, HBDs (both for neutral species and at pH 7.4) and net charge at pH 7.4 were used to set up a MPO model, which in turn was based on similar models described herein (59, 61, 64). The following parameters were reported for that model's performance; *Se* = 0.49, *Sp* = 0.97, *PPV* = 0.97, and *NPV* = 0.43 (64). Moreover, the authors state they prefer a high *Sp* (63). However, a *Se* as low as 0.49 (0.51 in the first abstract) means that a very large fraction of potential tracer candidates that are in fact brain permeable will be classified as non-permeable. Most reported models with moderate to good overall performance show a *Se* higher than 0.75 (see examples in Table 3), while still showing adequate *Sp*. Finally, a support vector machine model was developed as well, which reportedly showed better classification performance. This one, including several other machine learning models (no details are provided), are stated to be trained with 70% of the dataset and later evaluated with the remaining 30%, but no cross-validation was reported (64).

**Table 3:** Overview of a number of reported *in silico* prediction models of BBB-permeability, including information regarding the applied dataset and the performances.

**References**	**Method**	**Dataset**		**Validation**				
		**Training (CNS+/CNS–)**	**Test (CNS+/CNS–)**	***ACC***	***Se***	***Sp***	***CCR***	***MCC***
Crivori et al. (47)	PLS	46/64	49/71	0.75	0.90	0.65	n.r.	n.r.
Cruciani et al. (48)	PLS	46/64	35	n.r.	n.r.	n.r.	n.r.	n.r.
Doniger et al. (54)	SVM	154/120	25/25	0.82	0.83	0.80	n.r.	n.r.
Adenot et al. (49)	PLS	1,336/360	20/62	0.91	0.90	0.92	n.r.	n.r.
Li et al. (55)	SVM	276/139	n.v.	0.84	0.87	0.75	n.r.	0.65
Kortagere et al. (56)	SVM	186/165	n.v.	0.82	0.84	0.79	n.r.	0.64
Wang et al. (65)	kohNN	1,283/310	266/130	0.81	0.95	0.55	n.r.	n.r.
Wager et al. (59)	MPO	119/108^a^	50/50^b^	n.r.	0.76^b^	0.38^b^	n.r.	0.14^b^
Guerra et al. (53)	NN	96/12	74/4	0.82	0.85	0.25	n.r.	n.r.
Suenderhauf et al. (51)	DTI (CHAID)	65/55^c^	65/55^c^	n.r.	n.r.	n.r.	0.91	0.82
	DTI (CART)	65/55^c^	65/55^c^	n.r.	n.r.	n.r.	0.90	0.80
Martins et al. (27)	RF	1850^d^	120^d^	0.95	0.83	0.71	n.r.	0.74
Zhang et al. (61)	MPO	62/15^e^	n.v.	n.r.	n.r.	n.r.	n.r.	n.r.
Gunaydin et al. (60)	pMPO	299/366^f^	n.v.	n.r.	n.r.	n.r.	n.r.	n.r.
Wang et al. (66)	CON_ML	1,812/546	109/36 (201/36)^g^	0.95 (0.97)^g^	0.98 (0.99)^g^	0.83 (0.83)^g^	n.r.	n.r.
Gupta et al. (62)	MPO	270/720	50/50	n.r.	0.80	0.72	n.r.	0.78

a *No CNS–, instead CNS drug candidates were used and some of these had failed at a later stage*.

b *Model validation with a test set was performed by Gupta et al. (62)*.

c *A 10-fold cross-validation approach was used including random splitting of the dataset*.

d *The final ratio of CNS+/CNS– after Bayesian statistics to get an unbiased dataset was not stated. The test set was randomly withdrawn from the training set*.

e *CNS PET tracers not CNS drugs*.

f *The area under the receiver operation cure was 0.77*.

g *An extra validation was performed with 92 additional CNS+ compounds added to the test set*.

*PLS, partial least squares regression; SVM, support vector machine; kohNN, Kohonen's self-organizing neural network; MPO, multiparameter optimization; NN, neural networks; DTI, decision tree induction; RF, random forests; pMPO, probabilistic MPO; CON_ML, consensus machine learning model (consensus model built based on the prediction of five single models built on either SVM or k-nearest neighbor with different descriptors and resampling methods, see (66) for details); n.v., not validated with a test set; n.r., not reported*.

The abovementioned methods are simply highlighted to provide a general overview and get an awareness of the different methods available for *in silico* prediction of BBB-permeability. Table 3 shows an overview of a few predictions, what computational method they were based on, as well as details regarding the applied dataset and the model's performance during validation (when available).

## Evaluation and Comparison of *in silico* Predictions and Classification Rules Using a CNS Pet Tracer Dataset

**Figure 3:**
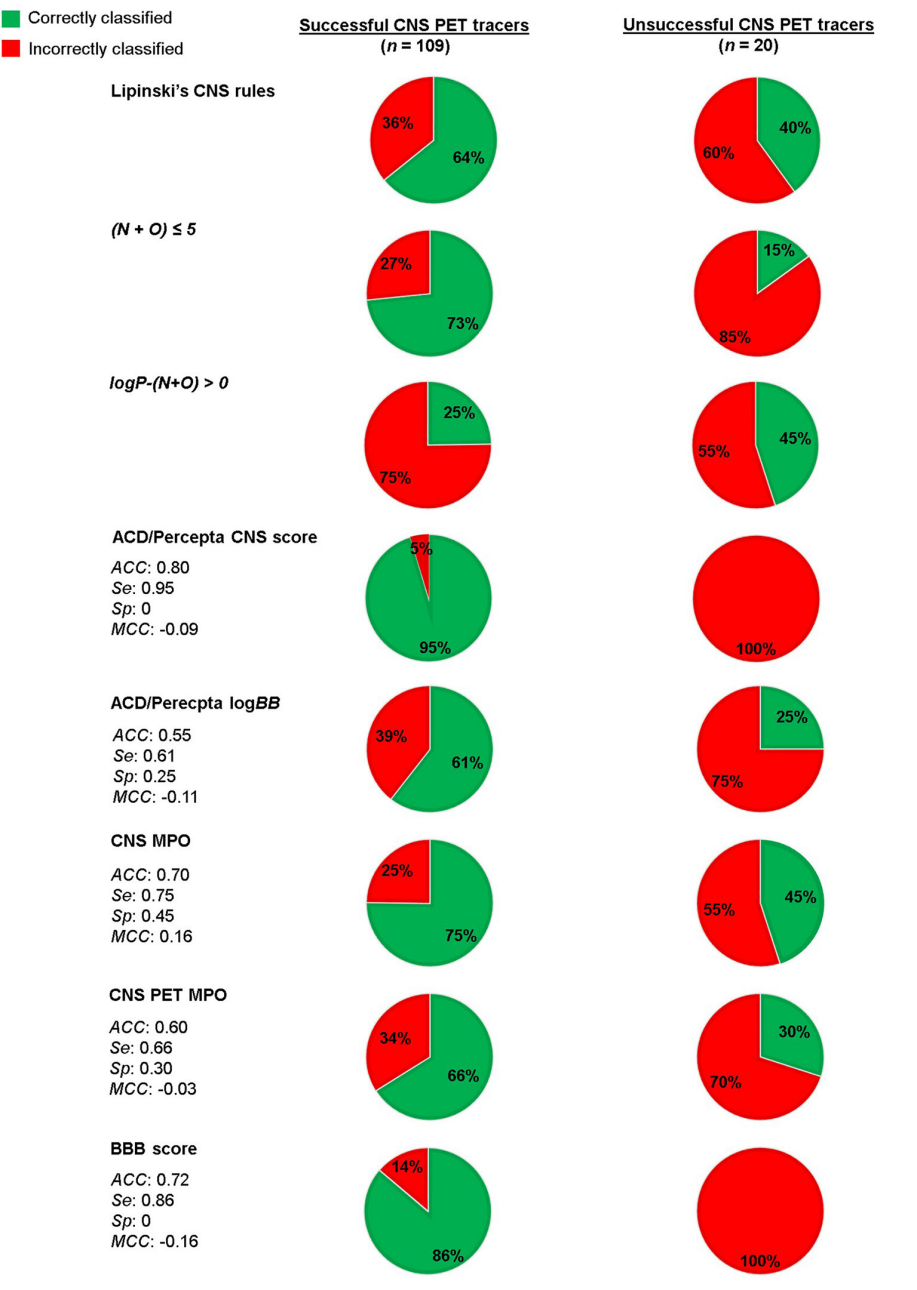
Evaluation of a CNS PET tracer dataset using a number of selected *in silico* predictions and classification rules.

The main reason behind the work toward the CNS PET MPO tool was that CNS PET tracers do not necessarily cover the same property space as CNS drugs and therefore need a dedicated *in silico* prediction tool (61). As previously highlighted, we believe this is most likely the case when ADME parameters are taken into account due to the differences in administered dose and the route of administration between PET tracers and drugs. On the other hand, for passive diffusion, a non-saturable mechanism, the property range should be the same for CNS PET tracers as for CNS drugs (17). As such, models relying on only passive diffusion should be able to predict BBB-permeability for CNS PET tracers as well. A small comparison of a few available models (both prediction of passive diffusion prediction and models aligned with ADME parameters) and rules of thumb using a combination of already reported CNS PET tracer datasets supported these suggestions. The model classifications are summarized in Figure 3. The dataset was based on the one used by Zhang et al. in the establishment of the CNS PET MPO model, but with the addition of a few more tracers (61, 67, 68). In total, it contained 109 successful CNS PET tracers and 20 unsuccessful ones. The latter are reported to cross the BBB, but are unsuccessful primarily due to high NSB (61, 67). Unfortunately, the number of unsuccessful CNS PET tracers in the dataset is limited since these are rarely reported in literature. Additional tracers in this category that are not crossing the BBB because of poor physicochemical properties, as well as tracers that are reported to have affinity for efflux transporters would have been preferred. Especially, in the context of evaluating a model that takes ADME parameters into account. The full CNS PET tracer dataset, with calculated properties and scoring can be found as Supplementary Material. The selected models for evaluation were CNS MPO, CNS PET MPO, the BBB score, CNS access score and log*BB* prediction from ACD/Percepta. In addition, the classification with the rules of thumb reported by Lipinski, and Norinder and Haeberlein, respectively, were reviewed. The following software packages were used to calculate the properties: MW (ChemDraw version 20.1.0.110, PerkinElmer Inc.); HBDs, HBAs, TPSA, log*D*_7.4_, and most basic p*K*a (ACD/Percepta, ACD/Labs release 2020.2.1, build 3,451, February 22, 2021, Advanced Chemistry Development Inc., Toronto, Canada); log*P* (Bio-Loom version 5, BioByte Corp., Covina, USA); BBB score (built-in function in ICM-Pro version 3.9-1c, Molsoft L.L.C., La Jolla, USA); CNS access score and logBB prediction (built-in function in ACD/Percepta, ACD/Labs release 2020.2.1, build 3,451, February 22, 2021, Advanced Chemistry Development Inc., Toronto, Canada). For the prediction models *ACC, Se, Sp* and *MCC* as a result of the screening were reported.

For Lipinski's rules for CNS drugs, 64% of the successful CNS PET tracers (70/109) adhered to the rules. For the unsuccessful tracers 60% was correctly discriminated by the rules to the CNS– category. Applying the rules by Norinder and Haeberlein on the dataset, resulted 73% of the successful tracers adhered to *N*+*O* ≤ 5 and only 3 of the 20 unsuccessful tracers were classified as CNS–. The Second rule, if log*P*-(*N*+*O*) is positive, then the log*BB* of the compound is positive. A positive value of log*BB* means the concentration in brain is higher than in blood. With this perception, only 25% of the successful tracers followed the rule. Although, 45% of the unsuccessful tracers had indeed a negative log*BB*, hence classified correctly.

Next, the dataset was scored using the CNS access score in the ACD/Percepta software. This is a composite score (Equation 8) of predictions of the brain/plasma equilibration rate [log(*PS* × *f*_*u,brain*_)] and the log*BB*, where *f*_*u,brain*_ is the fraction of unbound compound in brain tissue. The prediction model is partly based on the work reported by Lanevskij et al., in which a training set of 125 compounds with known log*PS* values have been fitted into a system of two non-linear equations. The descriptors taken into account include Clog*P*, HBDs, HBAs, McGowan's characteristic volume (*V*_*x*_) and ion fractions under physiological conditions (*f*_*i*_). The latter were obtained from p*K*a values of the strongest acidic and basic atom of the compound. The model was validated with an external test set and showed good prediction (*R*^2^ = 0.82 and *RMSE* = 0.49) (69). Not much information is provided on the prediction of the log*BB* parameter. However, the prediction is stated by ACD/Percepta to be based on a model trained on a dataset containing over 500 compounds with reported log*BB* values. Main descriptor determinants for prediction are Clog*P* and fraction unbound compound in plasma (*f*_*u,plasma*_). The CNS access score does only take into account passive diffusion.


(8) $$\text{CNS}\;\text{access}\;\text{score}\;=\text{ log}\left(\text{PS}\;\times \;{\text{f}}_{\text{u}}{,}_{\text{brain}}\right)\;+\;\text{logBB}$$

The CNS access score has the following thresholds: score < −3.50 for non-penetrant molecules, score −3.50 to −3.0 for weak penetrant molecules and score > −3.0 for penetrant molecules. In order to make a clear cut-off when screening the dataset, we considered also the weak penetrant compounds as penetrant, resulting in a threshold score > −3.50 for CNS+ compounds. The compounds classified as weak penetrants were all successful PET tracers. The prediction classified 95% of the successful tracers as CNS+ and all of the unsuccessful CNS PET tracers as well. This can be expected since the model only accounts for passive diffusion and the unsuccessful PET tracers in the dataset are so due to high NSB, an ADME parameter. The dataset was also screened separately in the ACD/Percepta log*BB* prediction module. For this the following classification was used: log*BB* > 0 for CNS+ and log*BB* ≤ 0 for CNS–. With these thresholds only 61% of the successful PET tracers were classified correctly, whereas 25% of the unsuccessful ones were classified as CNS–.

Finally, the CNS MPO, CNS PET MPO and BBB score were compared. The BBB score, which predicts passive diffusion, scored 86% of the successful tracers as CNS+ and all of the unsuccessful ones were scored as CNS+ as well. More relevant for the applied dataset are the CNS MPO and CNS PET MPO, which are aligned with ADME parameters as well. Interestingly, the CNS MPO, which is based on CNS drugs, showed both a higher *Se* (0.75 vs. 0.66) and *Sp* (0.45 vs. 0.30) compared to the CNS PET MPO. Bear in mind that the dataset is imbalanced, since unsuccessful PET tracers are under-represented. On the other hand, the dataset contains more unsuccessful compounds than the original datasets used to build the models. Even though the dataset does have its limitations, the results from the screenings indicate that the BBB score and the ACD/Percepta CNS access score, work well for predicting passive diffusion of the CNS PET tracers. The passive diffusion of several tracers in the dataset is supported by *in vitro* studies, where they showed values consistent with moderate to high passive permeability (61). When ADME parameters were included in the prediction, as for the two MPO models, a moderate fraction of the unsuccessful tracers were classified correctly. Interestingly, all of the unsuccessful CNS PET tracers were classified as strongly or extensively bound to plasma proteins in ACD/Percepta (shown in the CNS PET tracer dataset table provided as Supplementary Material). Since binding to plasma proteins is a contributor to the total non-specific binding, the outcome of the ACD/Percepta prediction further supports the non-specific binding issues reported with these unsuccessful tracers.

## Concluding Remarks and Future Perspectives

One of the most demanding challenges in both CNS drug discovery and CNS PET tracer development is to design compounds that can cross the BBB. Tools that can guide and facilitate this task at an early stage of the research and development phase is of utmost importance. Several *in vitro* assays have been reported for screening compounds for BBB-permeability (70–72). For instance, PAMPA (parallel artificial membrane permeability assay) is often used early on in the development process as it allows for high-throughput screening at a low cost (73). The disadvantage is that PAMPA only gives an indication of passive diffusion. In order to assess active influx/efflux more complex *in vitro* assays are needed (70–72). These, in turn, are not applicable for screening a large number of compounds. *In silico* BBB-permeability predictions are more efficient and cost-effective at an early stage and can be used both in screening campaigns and as a design tool during lead optimization.

Over the years, considerable progress has been made in setting up *in silico* BBB-permeability predictions. A number of different computational methods have been used, ranging from simple linear regression to more advanced machine learning methods. Some models are only predicting passive diffusion, whereas others take into account, e.g., efflux transporter liability. Thus far, there is only one prediction model available that is trained on CNS PET tracers (61). The disadvantage of this model is that it was trained with an imbalanced dataset, containing of 62 successful tracers and 15 unsuccessful tracers, a positive/negative ratio that does not reflect the reality. In addition, the model was not validated with an external dataset. Finding and setting up a good dataset is one of the main challenges in building any prediction model. For BBB-permeability prediction, it is difficult to find suitable CNS– compounds. Appropriate unsuccessful CNS PET tracers have to be included in the dataset depending on what the prediction model should actually assess. If not only passive diffusion, but also active transport, efflux and alignment with additional ADME parameters should be included, the dataset has to contain representatives from all those contributors. Furthermore, when selecting a model it is important to know what type of dataset was used for training of the specific model, the quality and size of this dataset and if the model was properly validated with an additional test set, as well as the outcome of the validation (values of statistical parameters such as *ACC, Se, Sp*, and *MCC*).

To refer back to the key points raised in the introduction, namely if a dedicated *in silico* model for CNS PET tracers is needed or if a model built on a CNS drug dataset can be used for predicting brain uptake of CNS PET tracers as well. In our opinion, if only BBB-permeability by passive diffusion is to be predicted, the favored property space for CNS drugs and CNS PET tracer is most likely the same, since the passive diffusion of a compound across the BBB is a non-saturable mechanism. Thus, an already established model trained on CNS drugs can be used and there are several well-performing models available for this purpose. In contrast, active transport across the BBB is a saturable mechanism, hence differences between tracers and drugs may occur. The field is constantly progressing with better models and larger datasets become available. Recently, a new dataset applicable for machine learning predictions was reported by Meng et al. This datasets contains 7,807 compounds, categorized into 4,956 CNS+ and 2,851 CNS–, but so far it has not been applied in training or validating any model (74). The fact that a model trained on CNS drugs can be applied for PET tracers as well is also what the analysis of the screening of the CNS PET tracer dataset indicated. The CNS access score in ACD/Percepta and the BBB score, which only predict passive diffusion classified 96 and 88%, respectively, as BBB-permeable of the entire dataset (successful + unsuccessful CNS PET tracers). The models that also relies on ADME parameters in the scoring, i.e., the CNS MPO and the CNS PET MPO displayed good to moderate *Se* and low *Sp*. On the other hand, it is difficult to draw conclusions regarding the performances of these models as they were trained on insufficient datasets and were not properly validated.

If other parameters, e.g., ADME parameters including active influx/efflux across the BBB should be considered in the prediction, an *in silico* model dedicated to CNS PET tracers would be more beneficial. In this context, two parameters of special interest are efflux transporter liability and NSB. To address the issue related to the latter, *in silico* predictions have been reported (10, 68). In the case of efflux transporters, classification models for derisking designing structures with efflux activities is a challenge. The most abundant efflux transporters in the human BBB are P-gp and BCRP (14, 15). For the former, several *in silico* models have been reported for classifying if a compound is a P-gp substrate or not (75–78). However, none of them show excellent accuracy. There are several challenges with setting up *in silico* models for this purpose. Firstly, all reported compounds are extremely structurally diverse due to the many binding sites in the P-gp structure (79). This makes quantitative structure-activity relationship (QSAR) models difficult to set up. Secondly, it has been suggested that compounds can be soaked up by P-gp within the membrane, meaning the diffusion rate across the membrane of a compound will have an effect as well (80). This, in turn, may explain the reported overlapping substrate specificity between P-gp and BCRP (81). Moreover, compounds with a slow passive diffusion may be more easily taken up, which in turn is something that can be considered when developing a prediction model. In respect to structure-based models, there are at the moment no high-resolution crystal structures available. Significant improvements will most likely be made when such structures will be resolved. The abovementioned reasons may also hamper the possibility to combine the assessment of efflux liability in a BBB-permeability prediction. Perhaps, it is even better two use separate predictions for a higher probability of success.

Finally, in order to set up a proper *in silico* prediction model (with consideration of ADME parameters) built for CNS PET tracers a suitable dataset is required. Collecting such a dataset will evidently be time-consuming and tedious, with respect to achieving appropriate size and chemical diversity. Only models that are built on carefully collected and adequate data can adapt to reality. Moreover, the dataset has to be chemically diverse, otherwise the basis of the QSAR hypothesis that chemically similar compounds tend to have similar activities has limitations as well (82). The structures in the dataset should also be drug-like. In the end, it is important to always keep in mind that “*all models are wrong, but some might be useful*” (*George E.P. Box*). A model will never represent the exact behavior of reality, but it can certainly be a useful tool.

## Author Contributions

All authors were involved in the preparation of the manuscript and approved the submitted version.

## Funding

EJLS received funding from the European Union's Horizon 2020 Research and Innovation Program as a Marie Sklodowska-Curie Individual Fellowship Under Grant Agreement No. 892572: Molecular Imaging of Microglia (MIM).

## Conflict of Interest

ADW is editor-in-chief of Nuclear Medicine and Biology. The remaining authors declare that the research was conducted in the absence of any commercial or financial relationships that could be construed as a potential conflict of interest.

## Publisher's Note

All claims expressed in this article are solely those of the authors and do not necessarily represent those of their affiliated organizations, or those of the publisher, the editors and the reviewers. Any product that may be evaluated in this article, or claim that may be made by its manufacturer, is not guaranteed or endorsed by the publisher.
